# Long-Term Persistence and Relevant Therapeutic Impact of High-Titer Viral-Neutralizing Antibody in a Convalescent COVID-19 Plasma Super-Donor: A Case Report

**DOI:** 10.3389/fimmu.2021.690322

**Published:** 2021-08-23

**Authors:** Mafalda De Rienzo, Maria Laura Foddai, Laura Conti, Chiara Mandoj, Stefano Iaboni, Ilenia Saladini, Concetta Castilletti, Giulia Matusali, Davide Roberto Donno, Luisa Marchioni, Stefania Ianniello, Angela Corpolongo, Maria Palange, Gennaro Ciliberto, Giulia Piaggio, Federico De Marco

**Affiliations:** ^1^Immunohematology and Transfusional Medicine Unit, Regina Elena National Cancer Institute – IRCCS, Rome, Italy; ^2^Clinical Pathology Unit and Cancer Biobank, Regina Elena National Cancer Institute – IRCCS, Rome, Italy; ^3^Virology and Biosecurity Laboratories, National Institute for Infectious Diseases “Lazzaro Spallanzani” IRCCS, Rome, Italy; ^4^Department of Infectious Diseases, National Institute for Infectious Diseases, “Lazzaro Spallanzani” – IRCCS, Rome, Italy; ^5^Department of Radiology, National Institute for Infectious Diseases, “Lazzaro Spallanzani” – IRCCS, Rome, Italy; ^6^Immunohematology and Transfusional Medicine Unit, San Camillo Forlanini Hospital, Rome, Italy; ^7^Regina Elena National Cancer Institute – IRCCS, Rome, Italy; ^8^Department of Research, Technological Innovation & Advanced Diagnostics, Regina Elena National Cancer Institute – IRCCS, Rome, Italy

**Keywords:** COVID-19, neutralizing antibodies, hyperimmune plasma, IgA, RBD/ACE2-binding inhibition test, case report

## Abstract

A convalescent, non-severe, patient with COVID-19 was enrolled as a hyper-immune plasma voluntary donor by the Immuno-Hematology and Transfusion Unit of the Regina Elena National Cancer Institute in Rome, under the TSUNAMI national study criteria. During a nearly 6-month period (May–October 2020), the patient was closely monitored and underwent four hyperimmune plasma collections. Serum SARS-CoV-2 (anti-S + anti-N) IgG and IgM, anti-S1 IgA, and neutralizing titers (NTs) were measured. Anti-SARS-CoV-2 antibody levels steadily decreased. No correlation was found between anti-S/anti-N IgG and IgM levels and viral NT, measured by either a microneutralization test or the surrogate RBD/ACE2-binding inhibition test. Conversely, NTs directly correlated with anti-S1 IgA levels. Hyperimmune donor plasma, administered to five SARS-CoV-2 patients with persistent, severe COVID-19 symptoms, induced short-term clinical and pathological improvement. Reported data suggest that high NTs can persist longer than expected, thus widening hyperimmune plasma source, availability, and potential use. *In vitro* RBD/ACE2-binding inhibition test is confirmed as a convenient surrogate index for neutralizing activity and patients’ follow-up, suitable for clinical settings where biosafety level 3 facilities are not available. IgA levels may correlate with serum neutralizing activity and represent a further independent index for patient evaluation.

## Introduction

Since the initial outbreak of a novel coronavirus emerged in Wuhan, China, in December 2019 (1), the severe acute respiratory syndrome coronavirus 2 (SARS-CoV-2) proved to be highly pathogenic and caused nearly 200 million cases of the related coronavirus disease 2019 (COVID-19), which was associated with high mortality, largely exceeding 4 million cases by 10^th^ July 2021 (2). Although widely investigated, the immune response to SARS-CoV-2 and its impact on the course of COVID-19 and outcome is still not fully understood. As successful protection against clinical disease and severe outcomes could be obtained by passive immunity, a great deal of research is focusing on neutralizing Abs and convalescent plasma (CP). Neutralizing Abs are known to be associated with protective immunity against secondary infection with SARS-CoV-2 in non-human primates (3, 4). Based on this rationale, COVID CP (CCP) has been proposed and used in severe, life-threatening cases and for compassionate use (5) and a number of human neutralizing monoclonal Abs have been shown to reduce the viral load in patients without an immune response at baseline (6), and are now available for clinical evaluation (7, 8). Currently, the use of hyperimmune plasma is limited by a number of critical issues, including little knowledge about the persistence of post-COVID-19 immunity in convalescents, lack of a consensus on a potential donor’s eligibility criteria, and a lack of standardized methods to quantitate the specific anti-SARS-CoV-2 humoral immunity. Regarding this last pivotal issue, a great number of different methods and technical approaches have been devised to measure the immune response of COVID-19, while the Ab kinetics and their pathological and prognostic relevance remain poorly understood (9–12). In addition, commercially available enzyme immunoassays could have a low accuracy to detect neutralizing titers (NTs) suitable for hyperimmune plasma donation, although they are endowed with high diagnostic accuracy to detect anti-SARS-CoV-2 Abs (13, 14).

Here, we report on a CCP donor whose NT persisted adequate to plasma transfer longer than 155 days after clinical healing, despite the total anti-SARS-Cov-2 IgG levels that steadily declined. Five patients with severe COVID-19 received hyperimmune plasma from this donor with beneficial effects. The *in vitro* receptor-binding domain (RBD)/ACE2-binding inhibition test (RABI test) proved a reliable surrogate assay for NT evaluation of candidate donor screening in the clinical setting. Finally, we deem it important to report that, as far as the reported case is concerned, anti-COVID-19-S1-protein IgA represents a relevant contributor to neutralizing activity.

## Methods

### Clinical Data Collection

Clinical information on six patients diagnosed with COVID-19 was obtained from clinical charts. One patient was admitted to the “A. Gemelli” University Hospital, in Rome, and became a plasma donor once convalescent. Five patients were admitted to INMI and transfused with plasma from the above-mentioned donor. Retrieved information (**Table 1**) included the following: demographic data, day of admission since symptom onset and presenting symptoms; temperature, the ratio of partial pressure arterial oxygen and the fraction of inspired oxygen (PaO_2_/FiO_2_), SOFA score, clinical complications, and outcome; laboratory data included white blood cell count, lymphocyte count, liver and kidney function, C-reactive protein, and IL-6; imaging data included chest CT scan; and, finally, treatment data included oxygen therapy, antiviral therapies, steroids, and heparin.

**Table 1:** Clinical information and management of the five patients who received plasma from the donor patient.

	Patient 1	Patient 2	Patient 3	Patient 4	Patient 5
Sex	Male	Male	Male	Male	Male
Age (years)	73	71	52	46	67
Weight (kg)	80	95	85	70	75
Comorbidities	None	Mental retardation	None	None	None
Time from disease onset to admission (days)	5	6	5	4	7
Admission to CP transfusion (days)	2	2	4	1	21
Number of CP units transfused	3	3	1	1	3 (1 from our CP donor)
MNT titre of CP units transfused	1 U 1:160 2 U 1:80	3 U 1:320	1:80	1:160	1 U 1:160
Complications prior to plasma transfusion	Moderate ARDS	Mild ARDS	Mild ARDS, micro-embolism	Mild ARDS	Moderate ARDS, micro-embolism, *Staphylococcus capitis* sepsis, and disseminated infection with cytomegalovirus
Complications (days after transfusion)	Candida sepsis (19)	Candida sepsis (15)	Polymicrobial sepsis (*P. aeruginosa, E. faecalis*) (8)	None	Entetrococcal faecalis sepsis (6)
Days of hospitalization (outcome)	33 (death)	31 (discharge)	22 (death)	33 (discharge)	38 (death)
PaO_2_/FiO_2_ just before transfusion	182	274	172	269	184
PaO_2_/FiO_2_ on day 12 post-transfusion	236	350	328	486	240
Lymphocyte count before transfusion	570	660	2280	1120	950
Lymphocyte count on day 12 post-transfusion	740	1170	2390	2000	760
SOFA just before transfusion	5	6	7	4	7
SOFA on day 12 post-transfusion	6	4	5	1	7

ARDS, acute respiratory distress syndrome; Mild ARDS, PaO_2_/FiO_2_ from 200 to 300; Moderate ARDS, PaO_2_/FiO_2_ from 100 to 200 (Clinical Management of Covid 19); CP, convalescent plasma; SOFA, Sequential Organ Failure Assessment.

### Hyper-Immune Serum Assessment

The TSUNAMI national study (TranSfUsion of coNvalescent plAsma for the treatment of severe pneuMonIa due to SARS-CoV2) is an open-label randomized controlled multicentre study promoted by Agenzia Italiana del Farmaco (AIFA – Italian Medicines Agency), Istituto Superiore di Sanità (ISS Istituto Superiore di Sanità) and coordinated by AIFA, ISS, and Foundation GIMEMA, Italy. The main plasma donor eligibility criteria are age 18–65 years, molecular or serological COVID-19 diagnosis, male gender or nulliparous female, no previous transfusion, and neutralizing Ab titer ≥1:160.

Evaluation of anti-SARS-CoV-2 IgG/IgM Abs was performed by the Clinical Pathology Unit of the Regina Elena National Cancer Institute (IRE). Blood samples were collected and separated by centrifugation at 2000 g for 20 min within 2 h of collection. The presence of IgG and IgM antibodies directed against both spike and nucleocapsid SARS-Cov-2 proteins was assessed by the MAGLUMI 2019-nCoV IgG and IgM chemiluminescence immunoassays on the fully automated MAGLUMI analyzer (SNIBE – Shenzhen New Industries Biomedical Engineering Co., Ltd, Shenzhen, China). The cut-off value was set at 1.0 AU/ml for both IgG and IgM; the overall reproducibility, declared by the manufacturer, ranged between 6.8% and 8.7%.

### Serum-Neutralizing Abs

Two parallel methods were used to assess the serum NT: The gold standard SARS-CoV-2 Micro Neutralization Test (MNT) and the indirect RABI test. The first method, implying the manipulation of hazardous viral stocks under highly demanding biosafety level 3 procedures and facilities, was performed at the INMI, the national referral laboratory for the TSUNAMI study. Serum samples were heat-inactivated at 56°C for 30 min and titrated in duplicate in seven twofold serial dilutions (starting dilution 1:10). Equal volumes of serum dilution and medium containing 100 TCID_50_ SARS-CoV-2 (strain: SARS-CoV-2/human/ITA/PAVIA10734/2020 obtained from European Virus Archive – Global [EVAg]) were mixed and incubated at 37°C for 30 min. Subsequently, sub-confluent Vero E6 cell monolayers were challenged with the virus–serum mixtures and incubated at 37°C in a 5% CO_2_ atmosphere. To standardize inter-assay procedures, high and low positive control (1:160 and 1:40 neutralizing activity, respectively) were included in each assay session. After 48 h, monolayers were scored for viral cytopathic effect by visual inspection under a low magnification light microscope. The highest serum dilution inhibiting at least 90% of the cytopathic effect was indicated as the MNT_90_.

The latter RABI test actually measures the interaction of the RBD of the Spike 1 protein with the cell receptor protein ACE2, a critical and limiting step in viral entry. Although representing an indirect surrogate index of viral infection, this method permits the rapid and convenient evaluation of neutralizing Abs in low-level biological containment environments, such as field activity, first-level clinical pathology settings, and outpatient clinics, where most patients with COVID-19 are likely to be cared for. For this purpose, the COVID-19 Spike-ACE2 Binding Assay Kit (Raybiotech Life, Inc., GA, USA.) was used. Briefly, serum serial dilutions ranging from 1:16 to 1:2048 were prepared in a final 100 µl volume containing a fixed amount of a standard ACE2 protein and loaded, in duplicate, onto an RBD-coated 96-multi-well plate. After overnight incubation at 4°C, the plate was rinsed four-times with 300 µl of washing buffer and challenged with goat anti-ACE2 Ab. After 1 h of incubation at 25°C, the plate was decanted, washed as above, and loaded with anti-goat horseradish peroxidase conjugate–Ab for 1 h at 25°C. The plate was then washed again as above and challenged with 100 µl of peroxidase substrate 3,3’,5,5’-tetramethylbenzidine, and after 40 min at room temperature for color development, the reaction was then stopped with 50 µl of 2.0 M sulphuric acid. The amount of reduced 3,3’,5,5’-tetramethylbenzidine was then evaluated by the A_450_/A_620_ measured by a Thermo Scientific Multiskan FC (Fisher Scientific Italia, Rodano, Italy). The total NT was then expressed as the reciprocal of the highest dilution yielding a 50% inhibition of ACE2/RBD binding activity (IC_50_) compared with the titer of the negative control, here represented by a pool of six human sera collected before April 2019. Non-linear sigmoidal standard curve interpolation (Prism 6.0 GraphPad Software, Inc. San Diego CA) was used to assign titers to sera dilutions with OD + SD, which did not match with the one of IC_50_.

### Anti-SARS-CoV-2 S IgA Detection

Specific anti-SARS-CoV-2 (COVID-19) Spike 1 IgA antibodies were assayed by the ELISA assay with the Human Anti-SARS-CoV-2 Virus (COVID-19) Spike 1 IgA Kit, provided by Alpha Diagnostic International (TX, USA) and used according to the supplier’s instructions. IgA titers were expressed as the reciprocal of the highest serum dilution yielding OD in the range of the internal positive control 1 plus two SDs. Non-linear sigmoidal interpolation to the internal standard curve was used to calculate the final titers of samples with OD values outside the above-mentioned range. Assay sessions were only validated provided both negative control (as above) and reaction blank OD values plus two SDs ranged below 20% of the internal positive control.

## Results

### Donor History

A 51-year-old man presented to the first aid emergency ward of University Hospital “A. Gemelli” in Rome (Italy) complaining of a fever (up to 39°C), dry cough, and progressive dyspnoea for 10 days. The patient had been administered a pharmacological treatment with antipyretics and clarithromycin 500 mg twice a daily, which remained without success and had tested positive to a PCR COVID-19 test. The patient had not traveled recently but had been in contact with two known SARS-CoV-2-positive patients (father and sister). On presentation, his body temperature was 37.4°C, blood pressure 130/70 mmHg, heart rate 82 bpm, respiratory rate 20 breaths/min, and oxygen saturation 88% on room air. The admission chest CT showed bilateral interstitial pneumonia without thromboembolic complications. The patient was administered a 15-day treatment with Prezista/ritonavir and hydroxychloroquine. He also received oxygen therapy with a Venturi mask 28% and prophylaxis with low-molecular-weight heparin. Following two SARS-Cov-2 RT-PCR-negative nasopharyngeal swabs, collected on days 18 and 21 after admission, the patient was dismissed and followed-up at home.

### Hyper-Immune Serum Assessment

Three weeks after the second negative viral test, the convalescent COVID-19 patient was invited by the Immuno-Hematology and Transfusion Unit of Regina Elena Institute (Rome, Italy) to participate to the TSUNAMI national study and was found to fulfill the recruitment criteria for the TSUNAMI study and blood quality and safety according to the Italian Ministerial Decree of November 2, 2015.

**Figure 1:**
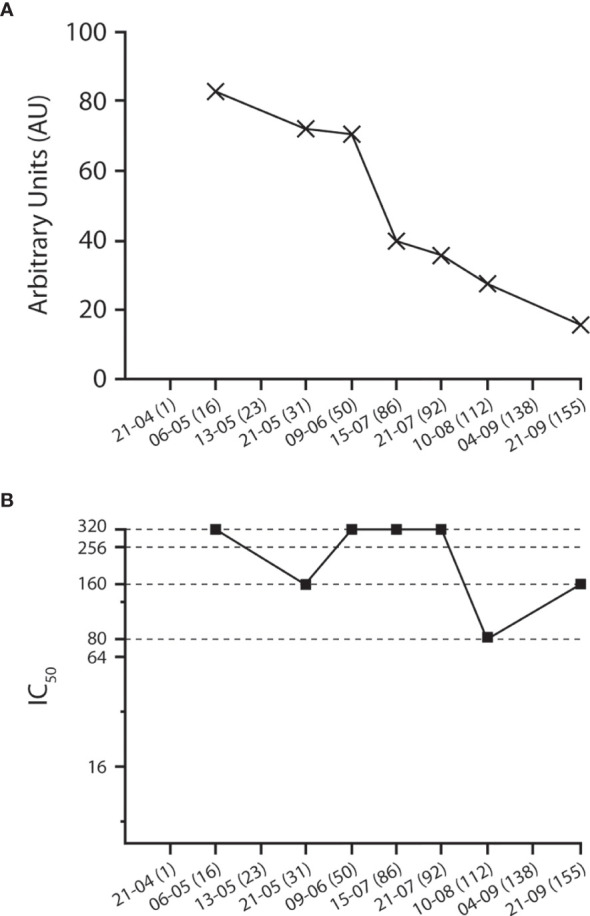
Kinetics of **(A)** anti-SARS-COV-2 IgG (anti-S + anti-N) and **(B)** neutralizing titer (MNT_90_). In parentheses, number of days elapsed since the second negative molecular nasopharyngeal swab.

Upon enrolment, the donor tested positive for anti-S + anti-N serum IgG with a titer of 82.86 AU/ml (**Figure 1A**), tested negative for IgM (i.e., 0.95 AU/ml), and had a 90% micro-NT (MNT_90_) of 1:320 (**Table 2** and **Figure 1B**).

**Table 2:** Serological test on the donor patient.

Date	IgG (anti-S + anti-N) (AU)	IgM (anti-S + anti-N) (AU)	MNT_90_(IC_90_)	RABI_50_(IC_50_)	IgA (anti-S + anti-N) (IC_50_)	Plasma donation
06/05/20	82.86	<1	1:320	1:512	1:1600	
21/05/20	72.14	<1	1:160	1:512	1:512	X
09/06/20	71.11	<1	1:320	1:432	1:288	X
15/07/20	39.08	<1	1:320	1:288	1:224	
21/07/20	35.58	<1	1:320	1:336	1:144	X
10/08/20	27.46	<1	1:80	1:192	1:144	X
21/09/20	15.27	<1	1:160	1:144	1:112	

IgG and IgM titers are expressed as chemiluminescent arbitrary units (AU). The cut-off value was 1.0 AU/ml for both IgG and IgM.

MNT titers are expressed as the reciprocal of highest serum dilution yielding a 90% inhibition of cytopathic effect (90% inhibitory concentration: IC_90_).

RABI and IgA titers are expressed as the reciprocal of highest serum dilution yielding a 50% reduction of maximal ELISA signal (50% inhibitory concentration: IC_50_).

Plasma was collected with a discontinuous flow cell separator, Haemonetics multicomponent collection system +, the volume of collected plasma was 600 ml, and each of the collected units was virus inactivated, aliquoted into three bags of 200 ml, frozen, and stored at the Immuno-Hematology and Transfusion Unit of San Camillo-Forlanini Hospital in Rome.

IgG, IgM, and MNT_90_ tests were repeated regularly and, thanks to the persistently high MNT_90_, the donor remained eligible for CP donation up to 155 days since enrolment. Throughout this period, in compliance with European recommendations and with his physical and medical condition (i.e., vascular accesses, plasma total proteins, and coagulation parameters), the donor underwent four successive plasma collections (**Table 2**).

**Figure 2:**
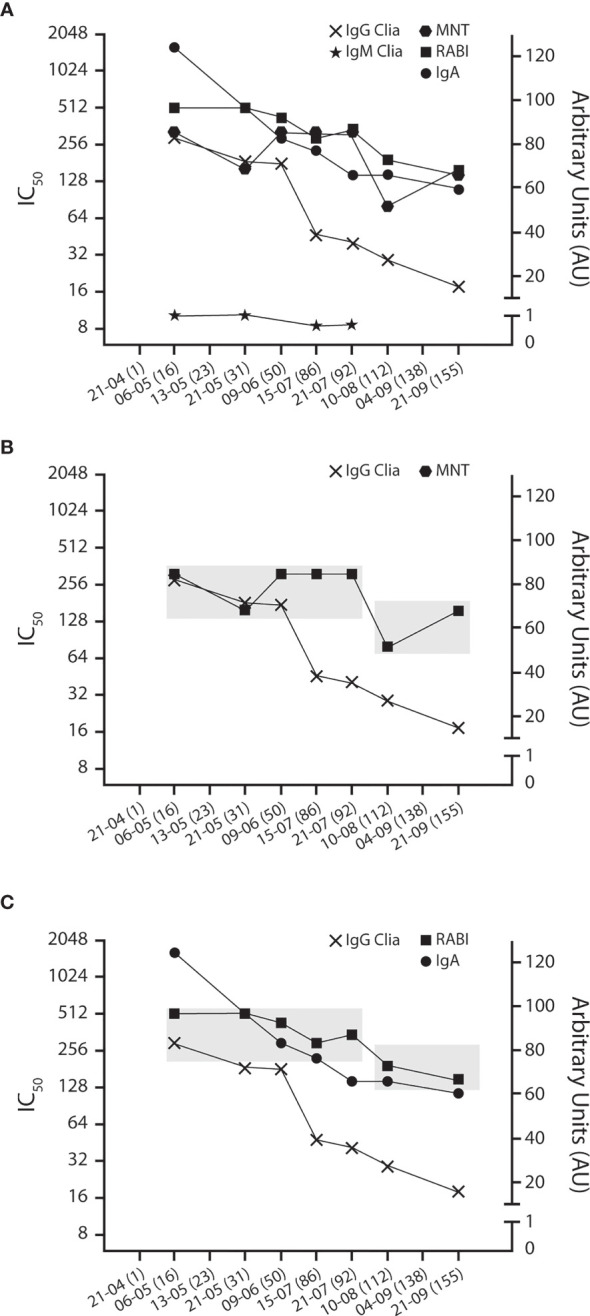
Antibody and neutralizing kinetics. **(A)** IgA, MNT, and RABI titers superimposed to anti-S + anti-N IgG and IgM. IgA, MNT, and RABI are expressed as reciprocal of dilution on a base 2 antilog scale (left “X” axis). IgG and IgM are expressed as AU on a linear scale (right “Y” axis). The chequered boxes highlight the stable profile of MNT **(B)** and IgA and RABI titer **(C)**, compared with anti-S + anti-N IgG.

In **Figure 2A**, the titers of anti-SARS-CoV-2 IgA, IgG, IgM, and neutralizing Abs (RABI) are superimposed to those of MNT. In agreement with data reported on the kinetics of serological response (15, 16), the anti-SARS-Cov-2 IgG titer steadily decreased from the initial 82.86 AU/ml down to 15.27 AU, while IgM was undetectable at any time point throughout the 155-day observation period. Conversely, the MNT_90_ remained stable. As a matter of fact, a twofold decrease in MNT_90_ was recorded from 6 May to 21 July 2020. Such a variation, however, remained isolated, and considering that at least a fourfold variation is needed for a dilution-based titer to be accepted as significant, it has to be regarded as a meaningless/irrelevant nominal fluctuation. Subsequently, a minor drop of MNT_90_ titer occurred during the period between 21 July and 21 September 2020.

As observed, there is no evident correlation between the steadily decreasing total IgG titer (“xs” on **Figure 2**) and the even level of MNT (solid squares on **Figures 2A, B**), indicating that RBD-targeted antibodies (i.e., those relevant to suppression of viral transmission and sterilizing immunity) may have different kinetics and longer persistence than those targeted to non-RBD epitopes. Such divergent kinetics have already been reported (15–17), and although no conclusive explanation has been proposed so far, it has to be considered in the enrolment and management of plasma donors.

Interestingly, the curve of the RABI test (open squares, **Figure 2C**) closely follows that of MNT, thus confirming the high neutralizing activity of the donor serum at any time point. Moreover, these results indicate that the RABI assay may represent a rapid and convenient surrogate test for the hazardous, labor-intensive, time-demanding MNT assay.

Anti-S1 IgA also showed a time-related progressive decrease. However, their titer, once again, closely matched the profile as obtained both by MNT and RABI test, suggesting that IgA may be a relevant contributor to the neutralizing activity (**Figures 2A, C**). Accordingly, their quantitative evaluation may represent an independent index of clinical evolution and outcome.

On 10 June 2020, because an intercurrent oropharyngeal/nasopharyngeal molecular swab tested positive for SARS-CoV-2 RNA by RT-PCR, plasma donation was temporarily suspended and then re-considered upon two successive negative swabs carried out 7 and 9 days later. As demonstrated in **Figure 1**, this molecular positivity was not associated with a change in the trend of decline of total IgG or with a boost in NT. Re-infection is a common event with most viral airway diseases. A few clinically evident cases have been exceptionally documented for patients with COVID-19 (18) and a persistent viral positivity has been reported for a minority of cases (16, 17), although its pathological meaning remains to be debated. In the case of our CCP donor, due to the lack of availability of the swabs collected from the initial COVID-19 infection, it was not possible to carry out comparative RNA sequencing to ascertain that there was a true re-infection. Nonetheless, the lack of increase in any of the measured antibody titer suggests that this was either a false-positive result or that a putative re-infection was rapidly resolved with no impact on the immune response (19).

### Convalescent Plasma Transfusion

Five male patients with COVID-19 (age range: 46–73 years) were admitted to “L. Spallanzani” National Institute for Infectious Diseases, Rome, Italy, diagnosed with COVID-19, and transfused with the plasma from the above-mentioned donor. The median time from symptom onset to hospital admission was 6 days (range: 5–7 days). The Sequential Organ Failure Assessment (SOFA) score at admission was between 3 and 4. Four patients (namely, patients 1, 3, 4, and 5) had no comorbidity, and three (patients 1, 2, and 3) were overweight with a BMI between 27 and 29. All patients received subcutaneous low-molecular-weight heparin; this was a prophylactic intervention in two cases and therapeutic treatment in three cases (the latter ones received concomitant intravenous methylprednisolone 0.5 mg/kg); three patients received intravenous remdesivir 200 mg on day 1 followed by 100 mg daily on days 2–5.

The patients received CP transfusion 7–26 days (median: 13) after symptom onset. The SOFA score was 5–7 immediately before transfusion and reduced, showing improvement, in two out of five patients after transfusion (patients 2 and 3) (**Supplementary Figure 1A**). The PaO_2_/FiO_2_ ratio ranged from 92 to 294 at admission, and from 172 to 182 immediately before CP treatment; they improved after CP transfusion for all patients (**Supplementary Figure 1B**). Lymphocytopenia improved after CP transfusion in four of the five patients (**Supplementary Figure 1C**). All patients presented bilateral ground-glass opacity and/or pulmonary parenchymal consolidation (on the chest CT scan), and two of them were carrying pulmonary micro-embolism immediately before CP transfusion (**Supplementary Figure 2**). After the transfusion, the pulmonary lesions in two patients (patients 2 and 4) markedly reduced (**Supplementary Figures 1A**, **3B**), while the remaining patients (patients 1, 3 and 5) had no significant improvement. During CP treatment, patients 1, 2, and 4 were on non-invasive ventilation, whereas patients 3 and 5 were on mechanical ventilation. Four patients (patients 1, 2, 3, and 5) had infectious complications and three of them eventually died due to sepsis (patients 1, 3, and 5) and never exhibited negative SARS-CoV-2 RNA, assayed by RT-PCR. Patient 2 was discharged after 31 days of hospitalization, and patient 4 after 33 days.

## Discussion

Here, we report evidence of the long-term persistence of high-titer viral-neutralizing Abs in a convalescent COVID-19 plasma donor. Although the total IgG titer steadily declined, neutralizing Abs measured by both MNT_90_ and RABI methods persisted at high neutralization titers for up to 155 days in the donor serum. Divergent kinetics between RBD-targeted and total anti-Abs have already been reported (15–17), and although no conclusive explanation has been proposed so far, it has to be considered in the enrolment and management of plasma donors. In light of these results, we can conclude that the anti-S/anti-N IgG test, which was originally devised to achieve the highest sensitivity in the screening of patients and asymptomatic carriers, appears not suited for patient follow-up and immunity surveillance. Interestingly, the surrogate RABI test readouts were in agreement with those generated by the gold standard MNT method. Thus, the RABI assay may represent a rapid and convenient surrogate for the hazardous, labor-intensive, time and technically demanding MNT test. This is particularly relevant in primary-level hospitals and care institutions devoid of biosafety level 3 facilities, where most COVID-19 patients are expected to be cared for. Our data point to a possible relevant role of IgA in the COVID-19 immune response. Actually, serum anti-S1 IgA levels showed a good correlation at each time point with neutralizing Abs measured either by MNT or by the RABI test.

Our result is consistent with data reported by Zeng et al. (20) showing that IgA from COVID convalescent patients had a stronger competitive capacity and a much higher specific neutralizing potency than IgG and IgM and suggests that anti-S1 IgA titer, at least in a part of cases, may be an independent serological index for patients evaluation and follow-up (20). Conversely, our finding is not consistent with recently published data showing a quick decay of IgA titers in CP donors (21). We have no hypothesis to propose for these conflicting data; however, a marked heterogeneity in immune response has been reported in the general population and among different CP donors (22, 23). Moreover, we recorded an extended IgA persistence at least in a part of other convalescent patients indicating that, at the present state of knowledge, no general rule response can be proposed to predict an outcome of each particular case, thus warranting detailed surveillance in potential plasma donors.

Nearly 3 months after the full symptomatic resolution, the donor tested positive for SARS-CoV-2 RNA. Such a finding is not unusual (17), and a number of explanations can be hypothesized besides re-infection. According to the WHO, a positive RT-PCR molecular test in the absence of new symptoms up to 100 days after the onset of the first infection is not considered reinfection, but a rather long shedding possibly involving non-viable virus, defective viral/sub-viral particles, non-infectious viral/sub-viral genomes, and viral/sub-viral subunits engulfed in phagocytic or immune cells. Available data do not allow to clarify this point.

In order to overcome the current CCP shortage (23), regulatory agencies have reduced the interval otherwise required for standard plasmapheresis donations (24, 25). In line with these guidelines, we performed plasmapheresis at minimum 15-day intervals. We observed that high NTs can persist for up to 155 days and probably longer, thus allowing for multiple donations carrying the opportunity to overcome the present CCP shortage.

In conclusion, a COVID-19 CP donor presented persistently high neutralizing anti-SARS-CoV-2 Abs, which definitely improved the clinical conditions of five patients with severe COVID-19.

The impact of reported findings is limited in that they are derived from a single patient serving as a plasma donor. Nonetheless, this case highlights several potentially interesting data. (i) At least in some cases, CCP can maintain neutralizing activity for a considerable time. Indeed, a comparable persistence of high RABI titers has been observed in a number of other cases, although ultimately not eligible for plasma donation (**Supplementary Figure 3**), thus suggesting that close monitoring is needed for precious plasma donors. (ii) CCP from a single donor improved the clinical conditions of five severely ill patients with COVID-19, pointing to a rather consistent therapeutic profile. Moreover, CCP, because of its polyclonal nature, is intrinsically resilient to RBD mutation, while the efficacy of monoclonal Abs may be dramatically reduced against the currently emerging viral variants (26). Thus, plasma super-donors may soundly contribute to relieving the current shortage of CCP. (iii) RABI may provide a rapid and convenient alternative to the highly hazardous, labor-intensive, viral neutralization test. (iv) IgA titer may represent an independent serological index for the evaluation and follow-up of patients.

## Data Availability Statement

The original contributions presented in the study are included in the article/**Supplementary Material**. Raw data are available at https://gbox.garr.it/garrbox/index.php/s/TTyNW88trUVuWVz.

## Ethics Statement

Ethical review and approval was not required for the study on human participants in accordance with the local legislation and institutional requirements. The patients/participants provided their written informed consent to participate in this study.

## Author Contributions

Conceptualization: GC, LC, FM, MR, MF, and GP. Data analysis and curation: FM, MR, and CM. Methodology: CC, GC, FM, MR, CM, and GP. Patients’ care: AC, DD, SIan, SIab, LM, MP, and IS. Investigation: FM, MR, CC, GM, CM, and GP. Resources: GC. Writing—original draft preparation. GC, FM, MR, and MF. Writing—review and editing: FM, MR, and GM. CT images: AC, DD, SIan, SIab, and LM. Graphs: FM and MR. Supervision: GC and FM. GP and FM equally contributed to this work. All authors contributed to the article and approved the submitted version.

## Funding

The study was supported by funds from the Italian Ministry of Health, Ricerca Corrente.

## Conflict of Interest

The authors declare that the research was conducted in the absence of any commercial or financial relationships that could be construed as a potential conflict of interest.

## Publisher’s Note

All claims expressed in this article are solely those of the authors and do not necessarily represent those of their affiliated organizations, or those of the publisher, the editors and the reviewers. Any product that may be evaluated in this article, or claim that may be made by its manufacturer, is not guaranteed or endorsed by the publisher.
