# Supplementary figures and images for: Long-Term Persistence and Relevant Therapeutic Impact of High-Titer Viral-Neutralizing Antibody in a Convalescent COVID-19 Plasma Super-Donor: A Case Report
Source: Front Immunol. 2021 Aug 23;12:690322. doi: 10.3389/fimmu.2021.690322 (PMC8419417; doi:10.3389/fimmu.2021.690322)

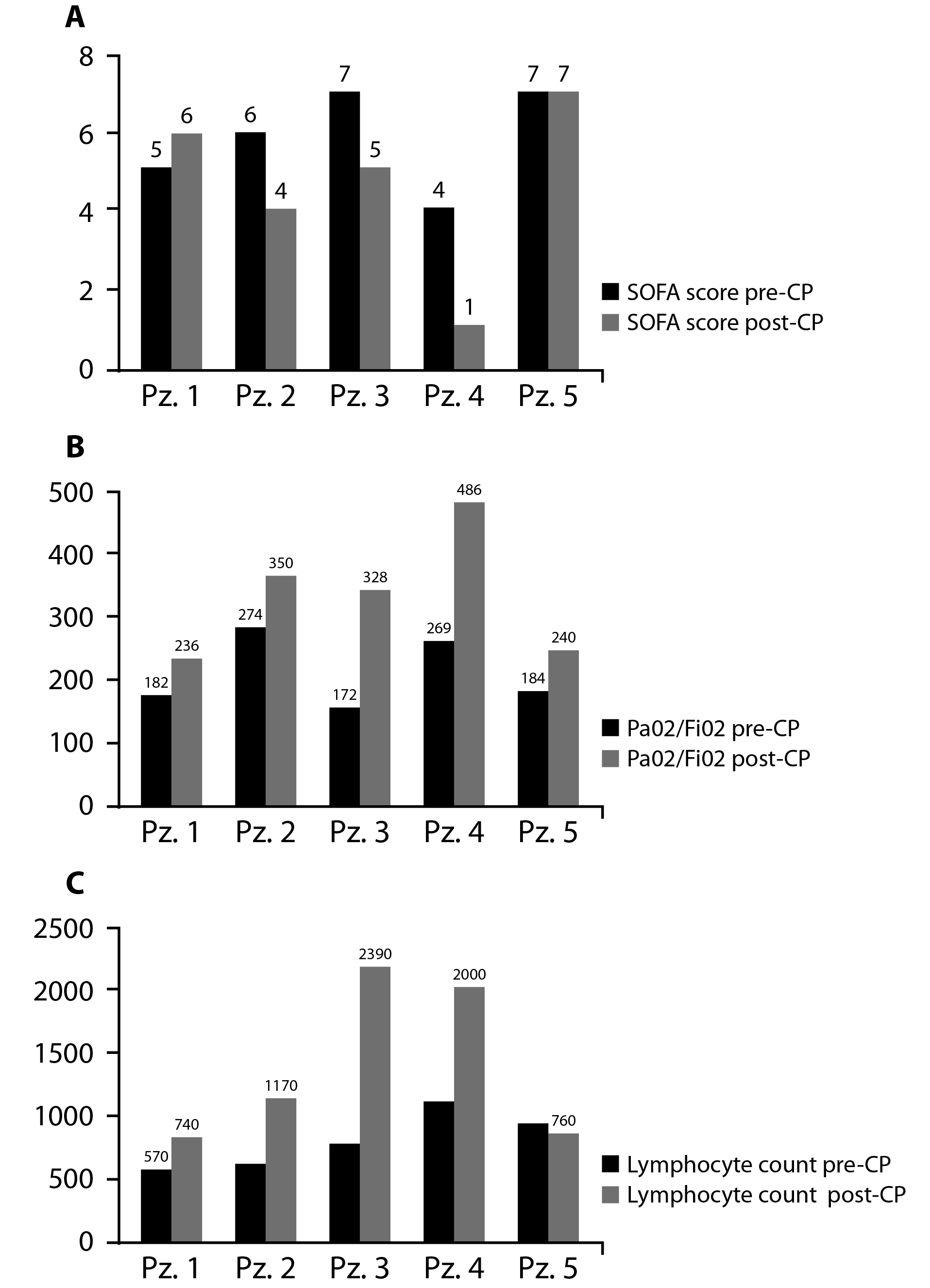

Supplement: Supplementary file 1 [file Image_1.jpg]

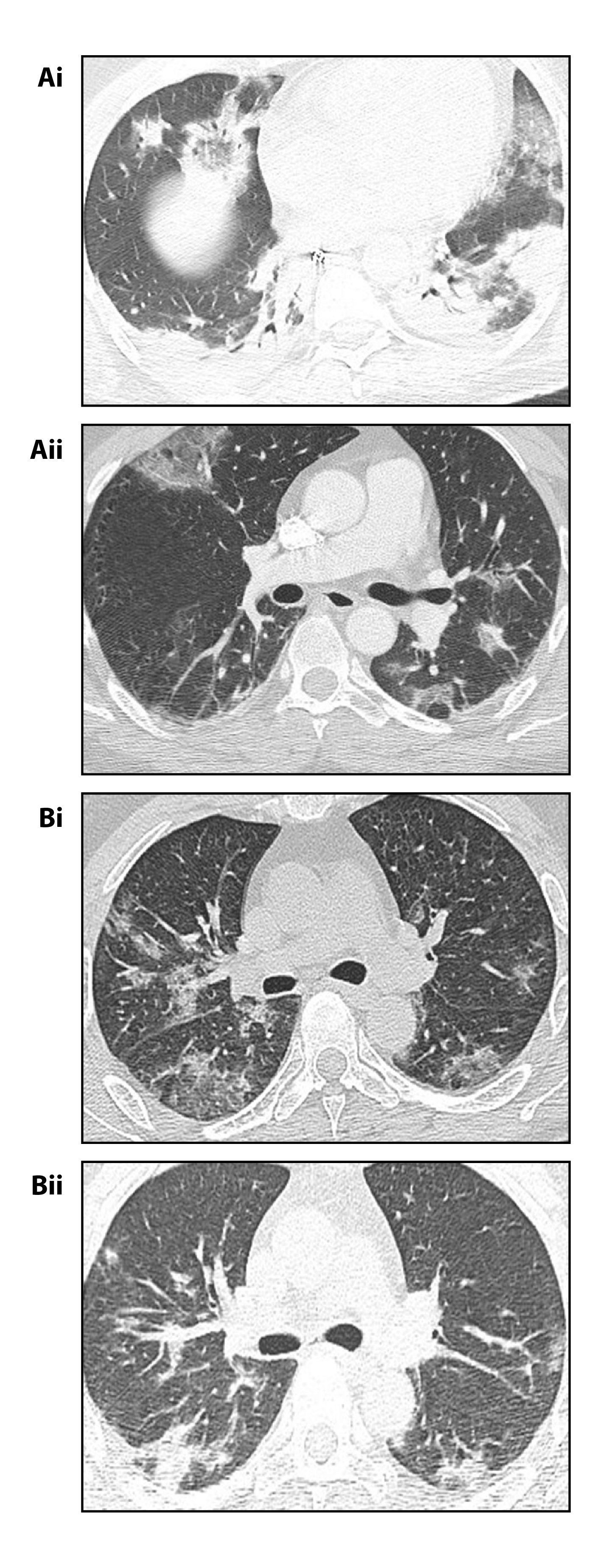

Supplement: Supplementary file 2 [file Image_2.jpg]

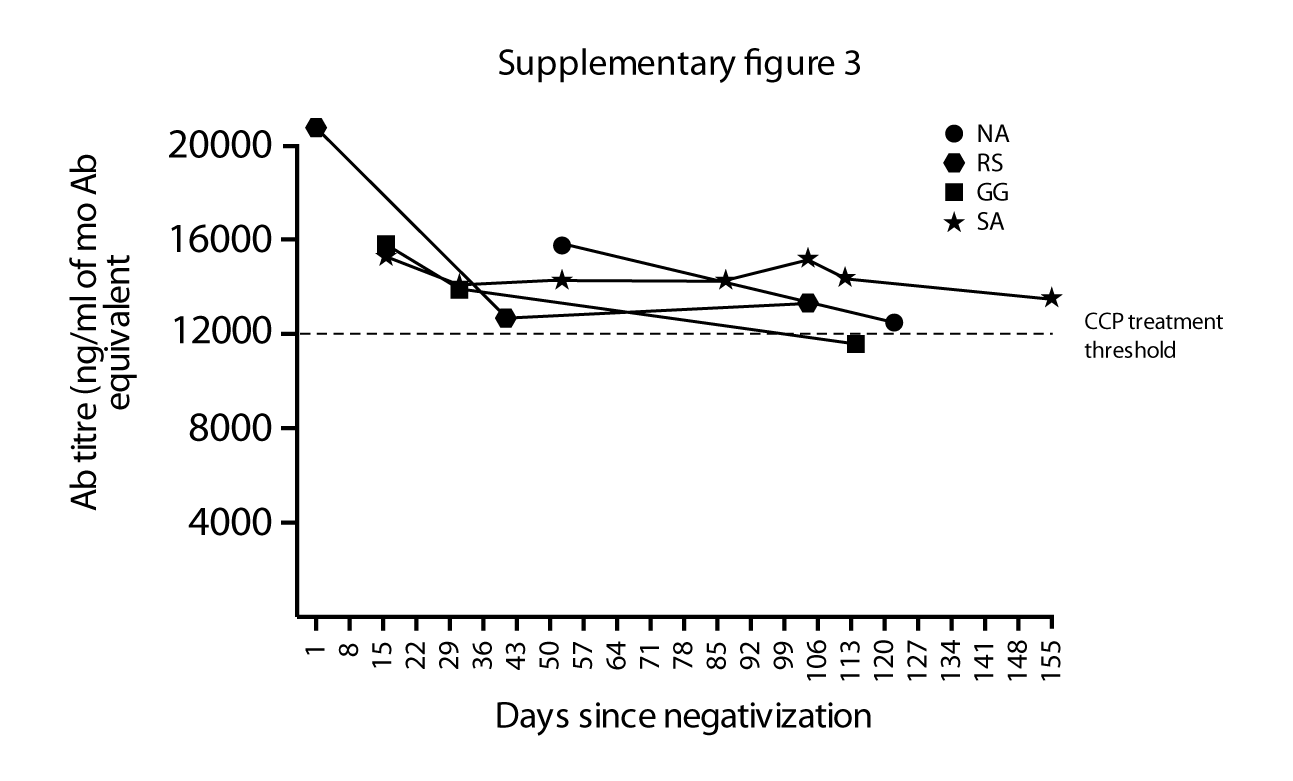

Supplement: Supplementary file 3 [file Image_3.tif]
